# Giant Right Atrium: An Extreme Structural Damage Case Due to Rheumatic Heart Disease

**DOI:** 10.7759/cureus.44898

**Published:** 2023-09-08

**Authors:** Clara L Voltarelli, Bruna Olandoski Erbano, Tiago Magalhães, Lucas H Olandoski Erbano, Talita Beithum Ribeiro Mialski, Rafael M Miyazima, Gustavo Lenci Marques

**Affiliations:** 1 Internal Medicine, Federal University of Parana, Curitiba, BRA; 2 Cardiology, Clinical Hospital Complex, Federal University of Parana, Curitiba, BRA; 3 Radiology, Federal University of Parana, Curitiba, BRA; 4 Medicine, Pontifical Catholic University of Parana, Curitiba, BRA

**Keywords:** valvular heart disease, giant right atrium, cardiac failure, atrial dilatation, rheumatic heart disease

## Abstract

Giant right atrium (RA) is a rare finding in adults. We report a case of a 53-year-old female with rheumatic heart disease (RHD) previously submitted to two mitral valve replacements. She presented at the emergency room with signs of heart failure. Image studies revealed gross cardiomegaly. Transthoracic echocardiogram showed torrential tricuspid regurgitation, with right chambers enlargement. At chest tomography, the estimated right atrium volume was 1,200 mL. The patient was treated with intravenous diuretics and multiple paracentesis, as well as referred to heart transplantation. Physicians should be aware of this extreme outcome, which can lead to life-threatening complications such as atrial fibrillation and thromboembolism.

## Introduction

Giant right atrium (RA) is a rare finding in adults. Such development can be an extreme consequence of rheumatic heart disease (RHD), since it stands as the leading cause of valvular heart diseases in low-middle income countries. The mechanism is most commonly due to mitral valve disease [1].

Despite efforts by the healthcare system in terms of prevention strategies, RHD is still an important cause of morbidity in young adults [2]. It is estimated that between 38.0 and 40.8 million people are affected by RHD globally [3], mainly aged between 20 and 50 years [4]. Rheumatic valvular diseases (RVD) may lead to extreme structural heart damage, such as secondary valve regurgitations, chamber enlargements and systolic ventricular dysfunctions, causing both left and right heart failure symptoms.

We present a case of a 53-year-old female presenting symptoms and signs of acutely decompensated heart failure, with RHD history and multiple hospital admissions due to medication nonadherence.

## Case presentation

A 53-year-old female patient presented at the emergency room with fatigue, lower extremities swelling and distended abdomen, which had worsened in the previous week. She also reported worsening of functional class (New York Heart Association (NYHA) III to IV), orthopnea, paroxysmal nocturnal dyspnea and irregular use of all medications in the same period.

Her medical history includes RHD, which required two mitral valve replacements: the first in 1990 (by a biological prosthesis) and the last in 2005 (by a mechanical prosthesis). Relevant history also included atrial fibrillation, hypertension and hypothyroidism. She was taking irregularly: warfarin 5 mg, levothyroxine 25 mcg, digoxin 0.25 mg, spironolactone 25 mg and furosemide 40 mg daily; carvedilol 25 mg and losartan 50 mg twice a day.

At admission, heart rate was 76 bpm (irregular rhythm) and blood pressure level of 115 x 64 mmHg. Physical examination revealed signs of hypervolemia such as jugular venous ingurgitation, lower body swelling, and distended abdomen. Heart valve auscultation revealed a metallic click on the mitral area and systolic murmur 3+/6, loudly heard at the tricuspid area. Initial laboratory findings showed an international normalized ratio (INR) level of 1.7.

The clinical features were interpreted as NYHA IV decompensated heart failure profile B, probably due to poor use of medication, since laboratory findings did not reveal inflammatory or infectious status, and even though cardiac rhythm was irregular, the ventricular response was adequate.

**Figure 1:**
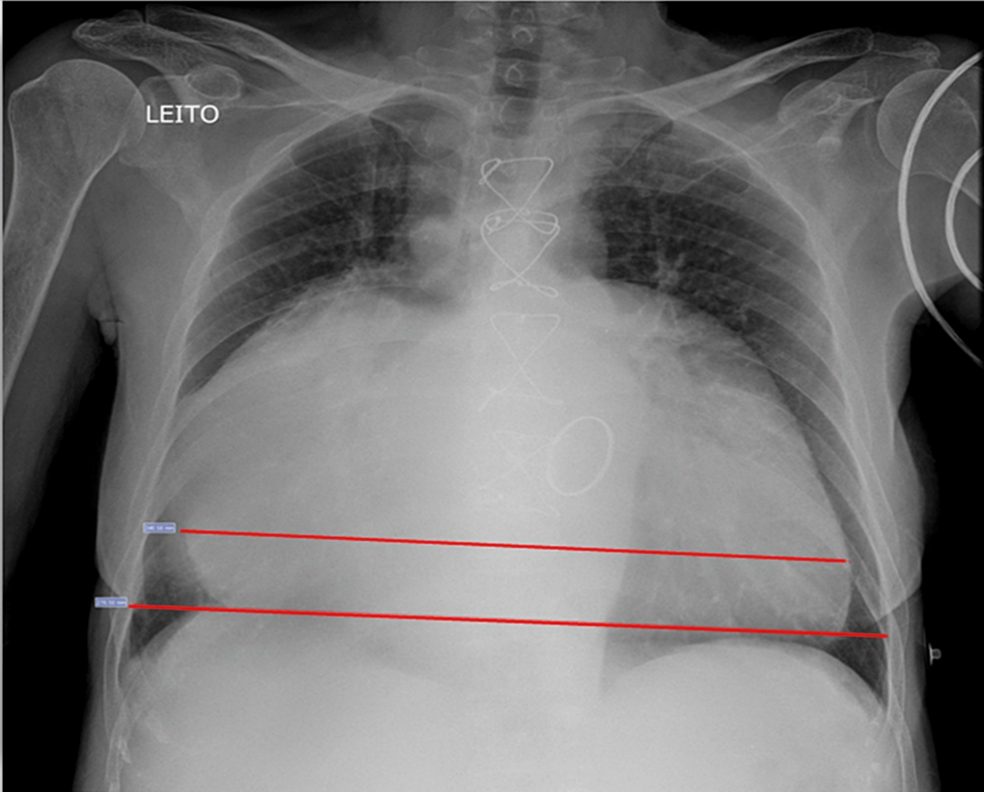
Chest X-ray taken upon admission showing severe cardiomegaly. Measured cardiothoracic ratio is 0.89.

Additional imaging tests were performed. A chest X-ray (Figure 1) showed severe cardiomegaly, with a cardiothoracic index of 0.89.

**Figure 2:**
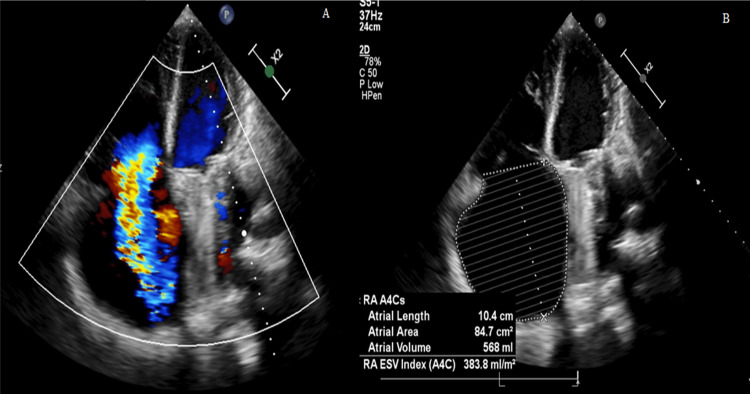
Transthoracic echocardiogram at four chambers view A) Color Doppler of torrential tricuspid regurgitation (vena contracta = 25 mm). B) Right atrium volume measurement.

Transthoracic echocardiogram (Figure 2) showed normal mobility and functioning of the mechanical prosthesis at the mitral position, normal left ventricular dimensions with moderate left ventricular systolic dysfunction due to diffuse hypokinesis (left ventricular ejection fraction (LVEF) of 35%) and left atrium enlargement (76 ml/m^2^). At right chambers, it was described: 1) severe right ventricular (RV) enlargement (67 mm basal), right ventricular systolic dysfunction due to diffuse hypokinesis (Fractional area change (FAC) of 21%); 2) torrential tricuspid regurgitation (lack of coaptation, one chamber hemodynamic mechanism); and 3) severe right atrium enlargement (380 ml/m^2^). The inferior vena cava measured 47 mm.

**Figure 3:**
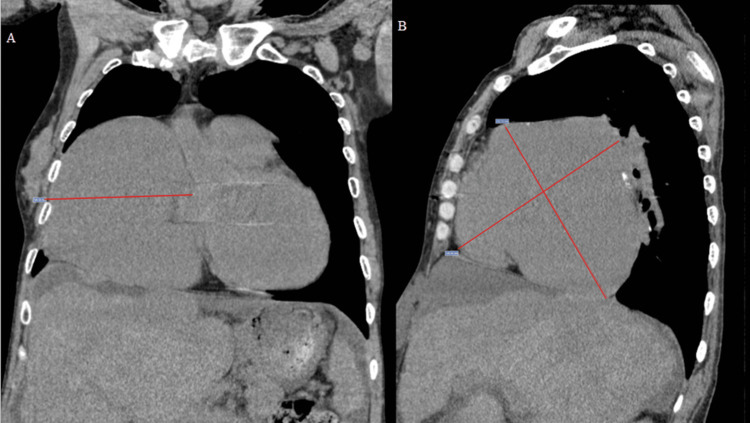
Non-contrast-enhanced chest CT: Coronal (A) and Sagittal (B) views. Estimated right atrium volume was 1,200.7 mL (through ellipsoid volume formula: 0.523 x height x width x length, which was 14.6, 10.4 and 15.2 cm, respectively).

Non-contrast-enhanced chest CT estimated the right atrium volume at 1,200.7 cm^3^ (through ellipsoid volume formula: 0.523 x height x width x length, which was 14.6, 10.4 and 15.2 cm, respectively). The pulmonary artery measured 3.2 cm (Figure 3).

Due to the worsening of LVEF compared to a 9-month previous echocardiogram, cardiac catheterization was performed and showed no obstructive atheromatous coronary disease.

Abdominal ultrasound showed signs of chronic hepatic disease, which was correlated with clinical and laboratory features and staged as Child-Turcotte-Pugh class B. The exam also showed severe ascites, supra-hepatic veins and proximal inferior vena cava severely enlarged.

**Figure 4:**
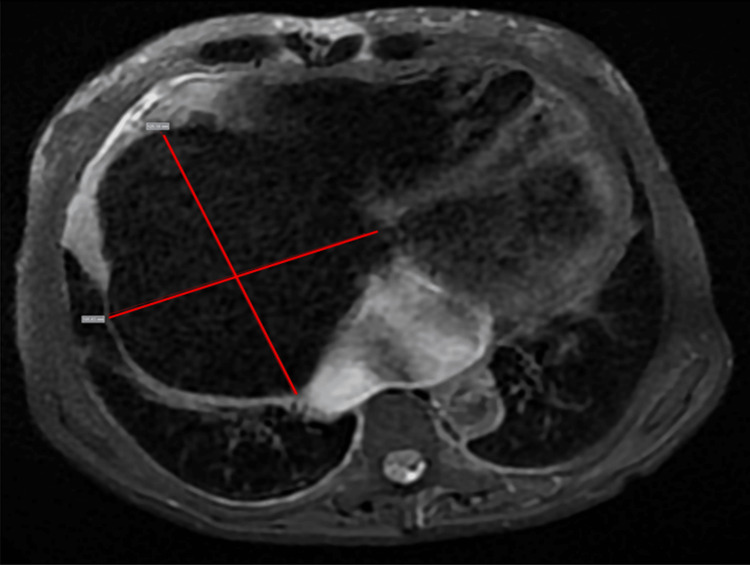
MRI scan. Axial T2-weighted scan with fat saturation shows a severely enlarged right atrium, measuring 12.5 x 10.5 cm (anteroposterior and transverse diameters).

In addition, an MRI (Figure 4) showed an enlarged right atrium, measuring 12.5 x 10.5 cm (anteroposterior and transverse diameters).

The patient was treated with intravenous furosemide and subcutaneous enoxaparin until her INR value achieved target between 2.5 and 3.5, due to mechanical mitral valve prosthesis.

Two paracentesis were performed, draining 300 mL and 1,800 mL, which resulted in significant clinical relief. Serum ascites albumin gradient (SAAG) calculated was 1.4 and ascitic protein above 2.5 g/dL, pointing towards transudate, probably related to congestion due to right heart failure.

Upon discharge, she reported improvement of dyspnea and a significant reduction of abdominal volume, although with residual lower body swelling (1+/4). She was instructed to modify pharmacological treatment taking spironolactone 100 mg daily and furosemide 80 mg daily, in addition to the previously prescribed medications, as well as reinforcement of the importance of adherence.

The patient was also referred to the Hepatology and Heart Transplant departments for further assessment and is currently awaiting heart transplantation.

## Discussion

Giant RA is more frequently found in children, due to congenital malformations, such as Ebstein’s anomaly [5,6]. In adults, this finding can be caused most commonly by valvular diseases, such as tricuspid valvular diseases (stenosis or regurgitation) or severe mitral valve diseases associated with pulmonary hypertension. Other causes described are cardiac infarction, pulmonary embolism and chronic pulmonary diseases [6].

Despite efforts by the healthcare system in terms of prevention strategies, RHD still remains as the main cause of valvular diseases in low-middle income countries. Mitral stenosis (MS) is the most common valvular dysfunction in RHD. Echocardiographic findings include thickening of the mitral leaflets and subvalvular apparatus, shortened chordae tendineae, commissural fusion, calcification, and restricted leaflet motion. Natural history of MS includes progressive structural heart damage, starting with left atrium (LA) enlargement and transmission of high LA pressures to the pulmonary vascular bed.

Thus, pulmonary hypertension is commonly found in patients with severe MS referred to interventions of the mitral valve. If not treated, right chamber damages can occur, such as enlargement, right ventricular systolic dysfunction and giant right atrium. In the RHD scenario, tricuspid regurgitation can be primary (rarely, due to RHV damage) or, most commonly, secondary to right chamber enlargement and, ultimately, lack of coaptation. In such cases, RA and RV function as one chamber [2], as presented by the patient reported here.

To this date, there is no medical treatment to prevent RHD progression. The only available measures intend to mitigate complications related to structural heart damage, which include atrial fibrillation, thromboembolic events, endocarditis and treatment of systolic heart failure. Medical treatments commonly include the use of beta-blockers, angiotensin converting-enzyme inhibitors, diuretics and warfarin [2,7], which were exemplified in the present case.

All severe and symptomatic valvular heart diseases require intervention (repair or replacement), which is proven to improve prognosis in the long term. The role of the heart team is crucial to define strategies and decide the best timing to intervene in valvular heart diseases, and to prevent extreme structural heart damage such as the giant RA reported here.

## Conclusions

Giant RA is uncommonly found in adults and can be caused by extreme structural heart damage due to RHD. This catastrophic complication increases the chances of complications such as atrial fibrillation and thromboembolism, which largely contribute to the disease burden. Physicians should be aware of this finding to better manage complications through early diagnosis and precise treatment.
